# An experimental protocol for *in vivo* imaging of neuronal structural plasticity with 2-photon microscopy in mice

**DOI:** 10.1186/2040-7378-5-9

**Published:** 2013-07-10

**Authors:** Christian Stetter, Markus Hirschberg, Bernhard Nieswandt, Ralf-Ingo Ernestus, Manfred Heckmann, Anna-Leena Sirén

**Affiliations:** 1 Department of Neurosurgery, University of Würzburg, Josef-Schneider-Str. 11, 97080 Würzburg, Germany; 2 Rudolf-Virchow-Center, University of Würzburg, Würzburg, Germany; 3 Institute for Neurophysiology, University of Würzburg, Würzburg, Germany

**Keywords:** 2-photon microscopy, Fluorescence, *In vivo* imaging, Neurons, Cranial window, Mouse model

## Abstract

**Introduction:**

Structural plasticity with synapse formation and elimination is a key component of memory capacity and may be critical for functional recovery after brain injury. Here we describe in detail two surgical techniques to create a cranial window in mice and show crucial points in the procedure for long-term repeated *in vivo* imaging of synaptic structural plasticity in the mouse neocortex.

**Methods:**

Transgenic Thy1-YFP(H) mice expressing yellow-fluorescent protein (YFP) in layer-5 pyramidal neurons were prepared under anesthesia for *in vivo* imaging of dendritic spines in the parietal cortex either with an open-skull glass or thinned skull window. After a recovery period of 14 days, imaging sessions of 45–60 min in duration were started under fluothane anesthesia. To reduce respiration-induced movement artifacts, the skull was glued to a stainless steel plate fixed to metal base. The animals were set under a two-photon microscope with multifocal scanhead splitter (TriMScope, LaVision BioTec) and the Ti-sapphire laser was tuned to the optimal excitation wavelength for YFP (890 nm). Images were acquired by using a 20×, 0.95 NA, water-immersion objective (Olympus) in imaging depth of 100–200 μm from the pial surface. Two-dimensional projections of three-dimensional image stacks containing dendritic segments of interest were saved for further analysis. At the end of the last imaging session, the mice were decapitated and the brains removed for histological analysis.

**Results:**

Repeated *in vivo* imaging of dendritic spines of the layer-5 pyramidal neurons was successful using both open-skull glass and thinned skull windows. Both window techniques were associated with low phototoxicity after repeated sessions of imaging.

**Conclusions:**

Repeated imaging of dendritic spines *in vivo* allows monitoring of long-term structural dynamics of synapses. When carefully controlled for influence of repeated anesthesia and phototoxicity, the method will be suitable to study changes in synaptic structural plasticity after brain injury.

## Introduction

Since its introduction in the 1990’s [1], 2-photon microscopy (2-PM) soon proved its enormous benefit for intravital imaging, especially in the field of neuroscience [2-8]. The possibility of penetrating tissue in depths up to 1 mm [5,7,9] and, therefore visualization of neural structures such as neurons, glial cells, and blood vessels led to new insights in developmental and degenerative neurobiology as well as neuronal plasticity after trauma, ischemia and inflammation [3,10] To obtain high-resolution *in vivo* images even in deeper areas of the brain (> 500 μm), highly ambitious surgical techniques and even use of fluorescence microendoscopy were developed [11-13]. In addition, combination of high speed, low power 2-PM calcium imaging with patch recodings allow monitoring of spine function [14] and long term neuronal network activity [15]. The availability of various transgenic mice expressing fluorescent proteins in particular cell types [2] enables selective observation of neurons, their axons, and dendrites in different layers [16], while simultaneously monitoring glial cells and blood vessels [4,10,17-20]. By creating a permanent entrance to the brain via a cranial window [21,22] in transgenic mice, even repeated and long-term 2-PM imaging became feasible [7,8,23]. The microstructure of neuronal tissue, e.g. dendritic spines and synapses, was shown to be a dynamic, highly delicate process of formation and elimination [16]. These new imaging methods will help us to better understand the role of synaptic plasticity after traumatic head injuries or degenerative disease.

## Materials and methods

For all experiments, we used male C57/Bl6 transgenic Thy1-YFP (H) mice expressing yellow-fluorescent protein (YFP) in layer 5 pyramidal neurons. All experiments required an appropriate animal experimentation facility and needed to be conducted in accordance with the laws and regulations of the regulatory authorities for animal care. The animal experiments presented here were approved by and conducted in accordance with the laws and regulations of the regulatory authorities for animal care and use in Lower Franconia (Regierung von Unterfranken, Würzburg, Germany; file number: 54–2531.01-20/07).

### Experimental Setup

**Figure 1:**
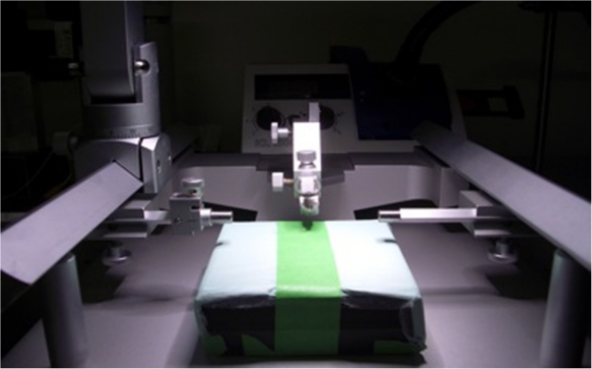
Stereotactic frame.

**Figure 2:**
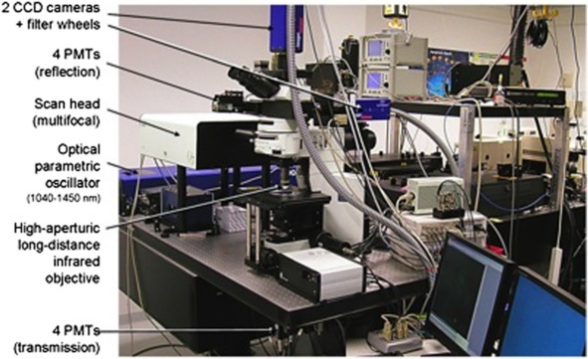
2-photon microscope.

**Figure 3:**
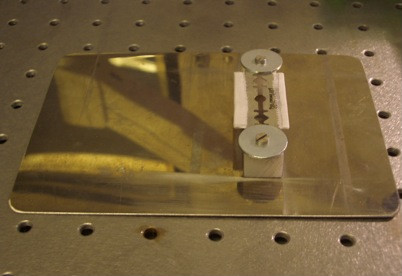
Custom-made head-holding device.

1. Operating microscope (Carl Zeiss AG, Jena, Germany).

2. Stereotactic frame (TSE, Bad Homburg, Germany, Figure 1).

3. Heating device.

4. 2-Photon microscope (Figure 2) with multifocal scanhead splitter (TriMScope, LaVision Biotec, Bielefeld, Germany).

5. Anesthesia unit.

6. Custom-made head holding device (Figure 3).

### Surgical instruments and materials

**Figure 4:**
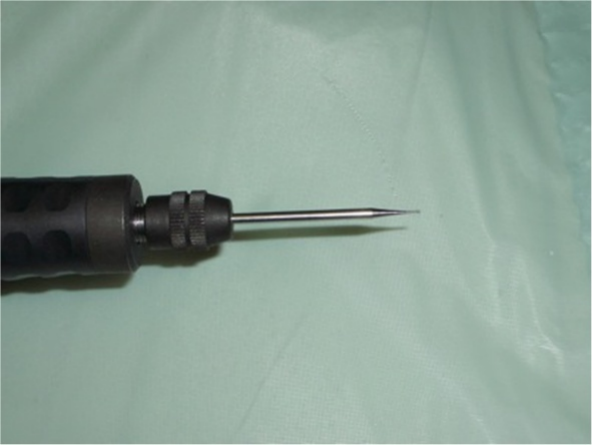
Diamond micro-drill tip.

1. Scalpel No. 15 (Aesculap, Tuttlingen, Germany).

2. Microsurgical blade (Surgistar #38-6961; Surgistar, Vista CA, USA).

3. Scissors (delicate curved sharp scissors; Aesculap, Tuttlingen, Germany).

4. Microdrill with diamond tip (diameter 1.5 - 3 mm; Figure 4).

5. Cyanoacrylate (Sigma-Aldrich Chemie GmbH, Steinheim, Germany).

6. Dental acrylic (Dentsply, York, PA, USA).

7. Custom-made cover slips (diameter 5 mm, thickness 1 mm).

8. Low-melting point agarose (1%, Sigma Type III; Sigma-Aldrich Chemie GmbH, Steinheim, Germany).

9. Sterile irrigation (e.g. sodium-chloride 0.9%, B.Braun, Melsungen, Germany).

10. Forceps (anatomical tips, straight or curved; Aesculap, Tuttlingen, Germany).

11. Needle holder (Aesculap, Tuttlingen, Germany).

12. Sterile suture material (Prolene 4.0, Vicryl 4.0; Ethicon, Norderstedt, Germany).

13. Anesthetics (xylazine/ketamine, isoflurane).

14. Cottonoids, swabs, gloves, and eye ointment (e.g. dexpanthenol).

## Methods and results

In the following section, we describe two different techniques to create a cranial window and illustrate the imaging setup. For surgery, all mice were anaesthetized with intra-peritoneal injection of 0.1 mg/g ketamine (Ketanest-S 25 mg/ml; Pfizer, New York, NY, USA) and 0.005 mg/g xylazine (Rompun 2%; Bayer Health Care, Leverkusen, Germany). The depth of surgical anesthesia was verified before starting surgery and the mouse head was fixed in a stereotactic frame (Figure 1). For *in vivo* imaging, mice were anaesthetized with isoflurane (Isofluran, Baxter, Deerfild, IL, USA) via a facial mask and the head was restrained in a custom-made head-holding device (Figure 3).

### Open-skull window

**Figure 5:**
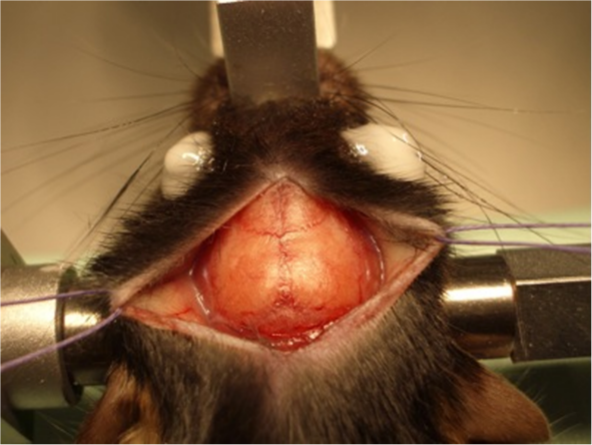
Exposed mouse skull.

**Figure 6:**
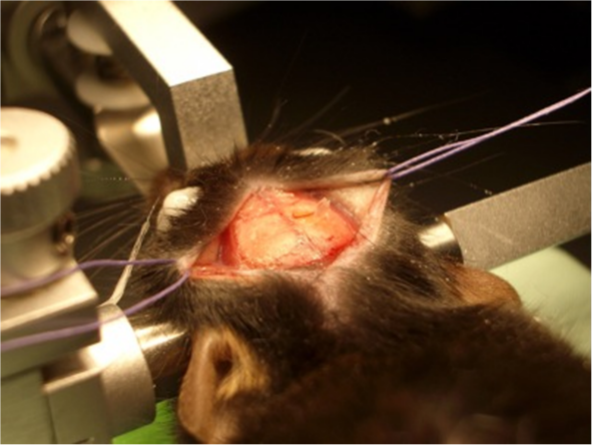
**Craniotomy over right parietal bone, bone flap still *in situ.***

**Figure 7:**
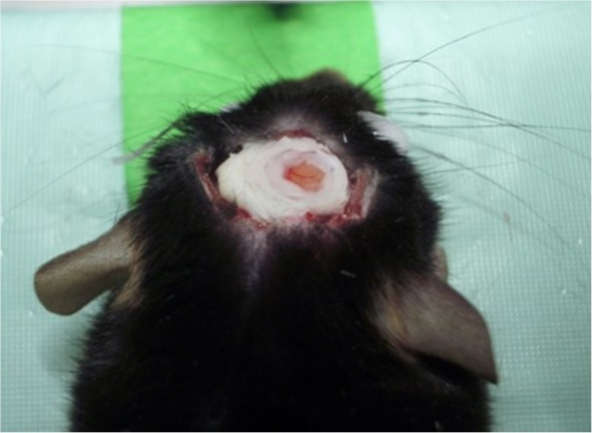
Glass cover slip fixed with cyanoacrylate and dental acrylic.

After fixation of the anaesthetized mouse in a stereotactic frame and application of eye ointment, a midline incision of the scalp was performed. Scalp and underlying periosteum were gently removed from skull bone with cotton swabs and the scalp was fixed laterally with two tack-up sutures (Figure 5). After localization of the region of interest (−1.5 mm bregma, 1.5 mm lateral), a craniectomy with the microdrill was carried out under the microscope and intermittent irrigation with sterile saline. Special care had to be taken before drilling away the last bone layer to avoid inadvertent injuries of the dura mater (Figure 6). Then, the exposed dura was covered then with fresh and sterile low-melting point agarose and a custom-made glass cover slip (diameter 5 mm, thickness 1 mm) was gently placed over the craniectomy and fixed with dental acrylic and cyanoacrylate (Figure 7). The crucial point here was to create a smooth agarose surface to prevent air bubbles between the agarose and the cover slip as well as averting fluid and sticky cyanoacrylate getting on the cover slip. Dental acrylic should be applied also on the exposed skull surface and the wound margins (the skin was not closed after the surgery). A strong micro magnet could be fixed in the dental acrylic for an alternative way to fix the head at the custom-made head holder instead of gluing it with cyanoacrylate repeatedly for long-term imaging (this could prevent cracking of glass cover while disconnecting the head from the head holder). After a recovery period of 14 days, imaging session could be started.

### Thinned-skull window

After restraining the mouse head in a stereotactic frame, the scalp was incised in the midline. Periosteum was softly separated from underlying bone with cotton swabs.

**Figure 8:**
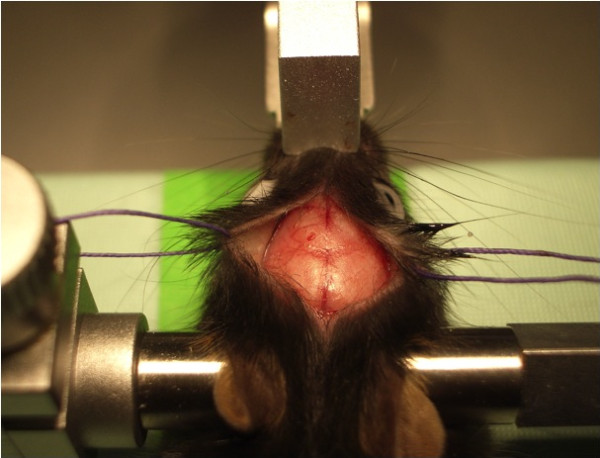
Thinned-skull cranial window.

The selected skull area (center of window 1.5 mm dorsolaterally of the bregma and the midline) was now carefully thinned in a circular area with the microdrill under the microscope until internal compact bone layer was reached (Figure 8). Generous irrigation is recommended for a clear view and to minimize the risk of heat-induced tissue injury. In the following step, the bone was continuously thinned in a cautious way with a microsurgical blade until the bone get so far thinned that cortex and vessels became visible. This procedure requires patience and dexterity because pushing and scraping to hard could damage brain and vessels, which leads to bleeding and inflammation or could even break the bone. Afterwards, one can start *in vivo* imaging immediately. Otherwise, the skin was sutured and the mouse was allowed to recover. For imaging sessions it is necessary to re-thin the skull with the microsurgical blades or to remove scar tissue.

### *In-vivo* imaging

**Figure 9:**
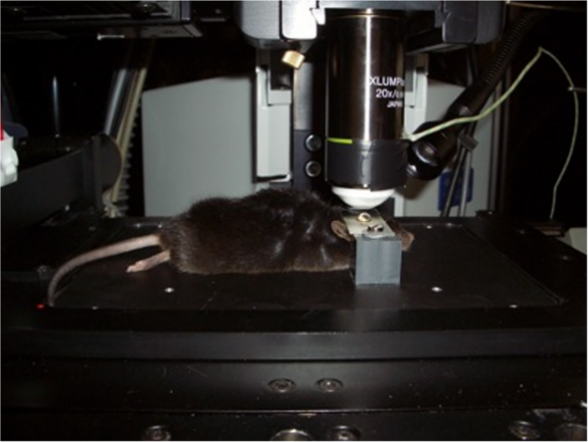
Mouse in head-holder under 2-photon microscope.

**Figure 10:**
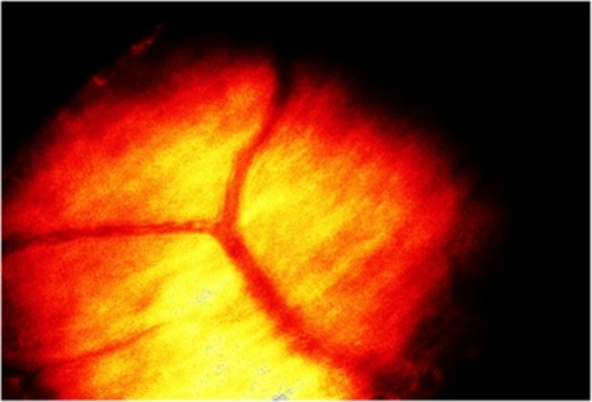
Meningeal blood vessels (video camera image).

**Figure 11:**
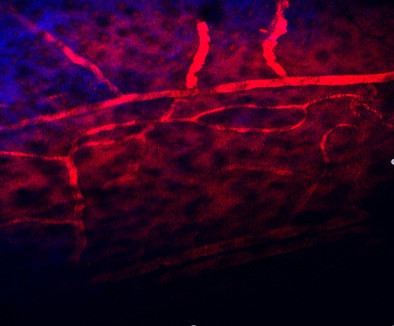
Cortical vessels under the 2-photon microscope.

**Figure 12:**
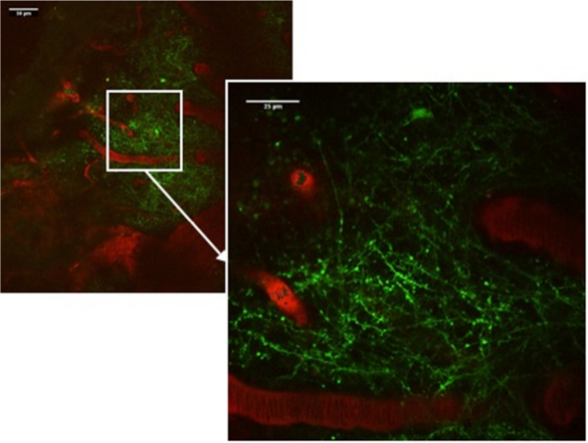
Rhodamine-dextran (red) filled cortical microvessels and Thy1-YFP labeled dendritic processes (green) in parietal cortex as viewed through a thinned skull cranial window.

For the imaging sessions, the mice were anaesthetized and the head was fixed in the custom-made head holder by gluing the skull to the triple razor blades with cyanoacrylate to reduce respiration-induced movement artifacts. The animal was placed on a heating plate under the two-photon microscope with multifocal scan head splitter (Figure 9). To facilitate relocation of the imaged area, a high-quality picture of the cortex surface with meningeal blood vessels was obtained with a CCD camera (Figure 10). The Ti-sapphire laser was then tuned to optimal excitation wavelength for yellow fluorescence protein (890 nm). Images were acquired by using a 20x, 0.95 NA, water-immersion objective (Olympus, Tokyo, Japan) in an imaging depth of 100–200 μm from the pial surface (Figure 11 and Figure 12). Two-dimensional projections of three-dimensional image stacks containing dendritic segments of interest were saved for further analysis. One of the difficulties in repeated imaging lies in preserving the cranial window in appropriate condition and to ensure exactly the same region of interest.

### Outlook

In this article, we provide a thorough methodological description of *in vivo* imaging of neuronal and vascular structures via two types of cranial windows. In experienced hands and with an established setup of two-photon microscopy, this method is a suitable tool for highly ambitious *in vivo* research, especially in the field of neurotrauma, neurodegenerative disorders, and neurovascular disease. The LaVision system was optimized for our application but the method is applicable for all two photon microscope systems. One of the advantages of the open skull method is that there is only one single surgery compared to multiple re-thinning procedures of the skull (and therefore multiple re-openings of the skin), an easier re-location of the same region of interest, and a higher penetration depth. However, the preparation of the open-skull window is demanding and bears a higher risk of dural tears and cortical injuries due to pressure or direct penetration. In addition, a damaged cover slip or opaque agarose layer could impair imaging results. Xu et al. reported a higher inflammation rate in neuronal tissue in the open-skull window with an increased turnover rate of dendritic spines [22]. Both models allow a “live” view on intracranial structures, not only on the surface of the brain, but even in deeper regions of neuronal tissue, and the possibility of long-term imaging.

## Competing interests

The authors declare that they have no competing interests.

## Authors’ contributions

CS carried out the surgical and imaging experiments, performed data analysis and drafted the manuscript. MHi designed and made custom-made devices and supported CS in performing imaging experiments. BN participated in the design and coordination of the study and supervised the experiments. RIE participated in the design of the study and edited the manuscript. MHe participated in the design and coordination of the study and supervised the experiments. ALS initiated, designed, supervised and coordinated the study and finalized the manuscript. All authors read and approved the final manuscript.
